# Association between Migraine and Quality of Life, Mental Health, Sleeping Disorders, and Health Care Utilization Among Older African American Adults

**DOI:** 10.1007/s40615-023-01629-y

**Published:** 2023-05-25

**Authors:** M. Bazargan, J. Comini, L.W. Kibe, S. Assari, S. Cobb

**Affiliations:** 1 Department of Family Medicine, Charles R. Drew University of Medicine and Science (CDU), Los Angeles, CA USA; 2 Department of Family Medicine, David Geffen School of Medicine UCLA, Los Angeles, CA USA; 3 Department of Urban Public Health, CDU, Los Angeles, CA USA; 4 Physician Assistant Program, CDU, Los Angeles, CA USA; 5 Mervyn M. Dymally College of Nursing, CDU, Los Angeles, CA USA

**Keywords:** Ethnic groups, Migraine, Headache, Quality of life, Morbidity, Chronic disease

## Abstract

**Purpose:**

This study examines the associations between migraine headaches, well-being, and health care use among a sample of underserved older African American adults. Controlling for relevant variables, the association between migraine headaches and (1) health care utilization, (2) health-related quality of life (HRQoL), and (3) physical and mental health outcomes was examined.

**Methods:**

Our sample included 760 older African American adults from South Los Angeles recruited through convenience and snowball sampling. In addition to demographic variables, our survey included validated instruments, such as the SF-12 QoL, Short-Form McGill Pain Questionnaire, and the Geriatric Depression Scale. Data analysis included 12 independent multivariate models using multiple linear regression, log transferred linear regression, binary and multinomial logistic regression, and generalized linear regression with Poisson distribution.

**Results:**

Having migraine was associated with three categories of outcomes: (1) higher level of health care utilization measured by (i) emergency department admissions and (ii) number of medication use; (2) lower level of HRQoL and health status measured by (i) lower self-rated health (ii) physical QoL, and (iii) mental QoL; and (3) worse physical and mental health outcomes measured by (i) higher number of depressive symptoms, (ii) higher level of pain, (iii) sleep disorder, and (iv) being disabled.

**Conclusions:**

Migraine headache significantly was associated with quality of life, health care utilization, and many health outcomes of underserved African American middle-aged and older adults. Diagnoses and treatments of migraine among underserved older African American adults require multi-faceted and culturally sensitive interventional studies.

## Introduction

Migraine headache is a common disorder characterized by recurrent, pulsating moderate to severe headaches with associated nausea, photophobia, and phonophobia [1]. Migraineurs may experience an aura prior to the onset of headache, commonly involving vision or sensory disturbances. It ranks among the top ten causes of disability worldwide and appears to be more prevalent among individuals of lower socioeconomic status [2]. Migraine accounts for approximately 1.5% of primary care visits annually.

The burden of self-reported migraine and severe headache in the US adult affects roughly 1 out of 6 Americans [3]. In 2018, the age-adjusted prevalence was close to 16% across all adults. Approximately 20% of women and 10% of men are affected [4]. Migraine carries a considerable physical, social, and economic burden [5]. Although the prevalence of migraine tends to decrease between 50 and 60 years of age, a significant number of older adults continue to experience migraine or have new-onset migraine [6]. The diagnosis of migraine in older adults may be more challenging and treatment options may be limited due to the presence of comorbid medical conditions [7, 8]. The clinical phenotype of migraine differs in the older adult patient group (compared to that of younger adults), with older adult migraine often occurring bilaterally, affecting the neck, presenting without pain, presenting with vegetative symptoms (i.e., loss of energy, anorexia) and being associated with “late-life migraine accompaniments,” referring to focal neurologic symptoms similar to that of an aura or transient ischemic attack [9, 10]. Given the decreasing prevalence of migraine with age, increased presence of risk factors for cerebrovascular disease, and the increased incidence of secondary headache conditions, older adult patients presenting with new-onset migraines may be subjected to additional diagnostic testing, such as neuroimaging, to rule out conditions such as cerebral infarction, transient ischemic attack, or hemorrhage.

Migraine has been noted to be comorbid with a number of conditions, including mental health disorders (depression, anxiety), cardiovascular disease, sleep disorders, and other neurological conditions, such as Alzheimer’s disease [11–14]. The prevalence of migraine within specific age strata in the USA were as follows: 19.4% (18–24 years), 19.0% (25–44 year), 19.4% (45–54 years), 14.0% (55–64), 9.5% (65–74 years), and 6.1% (> 75 years) [15]. However, there are limited epidemiological data about headache in elderly persons in the USA, especially those from minority backgrounds [16].

Examination of data collected by the National Health Interview Survey revealed that 15.5% of Whites and 14.45% of African Americans suffer from severe headache or migraine [17]. Migraine remains underdiagnosed and associated with worse outcomes among those of under-represented backgrounds and those who are underinsured or uninsured [18]. The American Migraine Prevalence and Prevention Study reported that migraine prevalence increased as lower household income decreased both for male and females [19]. While one study documented that migraine health care utilization, diagnosis, and treatment were low for African Americans, especially, for those who reported lower levels of trust and communication with doctors relative to Caucasians [20]. Another study found no major racial/ethnic differences in abortive or prophylactic treatment [21]. Indeed, reviewing current literature, Befus and colleagues in 2018 documented that African Americans were less likely to seek medical care and may not be receiving adequate care in comparison with their Caucasian counterparts [22].

Another extensive review of literature by Klenofsky and Shabas in 2019 showed that even though migraine is slightly less common in African Americans than Caucasians, African Americans are more likely to experience debilitating migraine headaches [23]. This study shows that African Americans have more frequent and severe migraines and the condition is more likely to become chronic [23]. Therefore, race-related disparities prevent African American migraine patients from accessing available treatments [24]. A recent study conducted by Amico and colleagues (2022) that included 61,453 hospitalized migraine patients revealed that African American, Hispanic, or Native American patients were more likely to have lower household income (*p* < 0.001), whereas Caucasian patients had a higher income [25]. However, studies on race-related gaps and possible healthcare inequalities in people with headache disorders are virtually non-existent [26]. Understanding potential race-related disparities in migraine patients may inform the development of culturally contextualized health care policies and interventions that are more likely to reduce or eradicate race-related headache disparities [24].

A health equity approach to migraine research and treatment requires examining underlying, intersecting social and structural factors as well as biological and behavioral contributions [22]. Therefore, continued research regarding disparities in migraine headache medicine is urgently needed [27]. This study examines correlates of migraine headaches among a sample of underserved older African American adults. Controlling for age, gender, education, marital status, satisfaction with and access to medical care, and number of major chronic conditions, the association between migraine and the following outcome variables are examined: (1) health care utilization; (2) health-related quality of life (HRQoL) and self-rated of health; and (3) physical and mental health outcomes.

## Methods

### Study Population

We recruited 760 adults from senior housing centers, senior centers, faith-based organizations, and apartment complexes in the South Los Angeles community. We included participants who self-identified as African American aged 55 years or older and committed to remaining in the area for the next year. Recruitment was completed during regular office hours, usually in the dining or day room areas. The Service Planning Area 6 (SPA6) of Los Angeles (LA) County is home to over one million residents. This area of LA County is disproportionately burdened by health disparities compared to all of Los Angeles County [28]. A good example is the hospitalization rate for heart failure, which is significantly higher for SPA6 compared to the rest of Los Angeles county (rate700/100,000 for South LA versus 350/100,000 LA County). It is disparities such as these that are tied to the objectives of this project. All the participants provided signed informed consent. Charles R. Drew University of Medicine and Science’s Institutional Review Board (IRB) approved the study protocol.

### Measurements

#### Survey Instruments

Along with standard items that measure demographic variables, our survey included validated instruments to document HRQoL [29], self-rated health status and health care access, the Short-Form McGill Pain Questionnaire-2 (SF-MPQ-2) [30, 31], and Geriatric Depression Scale (GDS) [32, 33].

#### Demographics Characteristics

Age, gender, educational attainment, marital status, and race/ethnicity were the demographic covariates in this study. We operationalized education attainment as the number of years of school attendance with higher values indicating more years of education. Marital status was categorized as a dichotomous variable: married or living with a partner (yes/no).

#### Financial Strain

Five items were used to measure financial strain. Participants were asked about the frequency of their inability to perform the following actions in the last 12 months: (1) purchasing enough food for their family, (2) buying clothes they deemed necessary, (3) paying rent or mortgage, (4) paying monthly bills, and (5) making ends meet. Responses were recorded on a 5-point scale, with options ranging from 1 (never) to 5 (always). We computed a total score for “financial strain” by averaging the responses across the five items, with scores ranging from 1 to 5. Higher scores indicated greater financial difficulty. These items are in line with Pearlin’s list of chronic financial challenges faced by low SES individuals and showed high internal consistency (Cronbach alpha = 0.92) [34].

#### Satisfaction with and Access to Medical Care

Six items were used to measure this variable. Participants were asked (1) In the last 12 months have you needed to see a doctor but could not see one? (2) Overall, how difficult is it for you to get medical care? (3) How difficult would it be for you to get a routine physical exam? (4) How difficult is it for you to visit a doctor when you need medical care? And (5) overall, how satisfied are you with how available medical care is for you? And (6) overall, how satisfied are you with how available medical care is for you? Using principal component analysis, primary factors underlying this 6-item instrument were identified. Varimax rotation produced two distinct factors, explaining almost 63 of the variance. The first factor explained 36% of the variance while the second factor explained 27% of the variance. All items had primary loadings over 0.5, and none of the items had a cross-loading above 0.24. The first factor was associated with the 3 items that measured perceived difficulty accessing medical care and the second factor was associated with three items that measured satisfaction with care.

#### Disability Status

Disability status was assessed as a dichotomous variable with a single item asking participants whether s/he is disabled (yes/no).

#### Health Care Utilization

Participants were asked (1) how many times they had utilized Emergency Department (ED), (2) stayed overnight at a hospital as a patient, or (3) visited a provider at their office in the last 12 months.

#### Major Chronic Conditions

Participants reported whether they had been diagnosed with the following medical conditions: high blood pressure, diabetes mellitus, heart-related conditions, cancer, stroke, chronic obstructive pulmonary disease (COPD), or a sleep disorder.

#### Self-Rated Health Status

This variable was measured by the question: “In general, would you say your health is (1) excellent; (2) very good; (3) good; (4) fair; and (5) poor?” Because self-rated health is differently shaped by social determinants across ethnic groups, this item is often used in national surveys and predicts mortality risk in ethnic groups.

#### Pain Severity

Pain was measured using the Short-Form McGill Pain Questionnaire-2 (SF-MPQ-2) [30, 31]. Participants self-reported the level to which they experienced each of four subscales derived from 22 pain items experienced in the past week. The form uses an 11-point numeric rating scale. Psychometric properties of the SF-MPQ-2 among non-Hispanic and Hispanic White patients with pain show that this measure is comparable across these 2 ethnic groups [35].

#### Health-Related Quality of Life (SF-12)

The SF-12 health survey measures include two subscales that provide an indication of physical and mental functioning and global health-related quality of life [29]. The SF-12 is a multipurpose, 12-item selected from the SF-36 Health Survey [36]. The SF-12 is a validated measure for assessing health status among low-income minority populations [37]. We computed physical and mental health composite scores (PCS & MCS) using scores of the twelve questions. The composite scores range from 0 to 100, where a zero score implies the lowest level and 100 indicates the highest level of health. PCS and MCS scores tend to vary over the life span for different age groups: PCS tends to decrease with age, while MCS tends to increase. The mean difference score specific to an individual’s age reflects the deviation of their score from the average score of their age group. If an individual scores higher than the mean, it suggests better health status compared to most others in the same age group. On the other hand, scores lower than the mean suggest poorer health status compared to most others in the same age group [38].

#### Depressive Symptoms

Depression was evaluated using the 15-item short Geriatric Depression Scale (GDS) [32, 33]. Responses were on a binary yes/no scale. The total score was computed by summing up the responses, yielding a possible range of 0 to 15, where higher scores indicated more severe depressive symptoms. The GDS-Short form has been widely adopted in both clinical and community contexts for measuring depression among older adults due to its excellent reliability and validity [32, 33].

#### Medication Use

We examined all medications by the drug inventory method. Participants were asked to bring all over-the-counter and prescribed medications that they had taken within two weeks prior to the interviews. The interviewer transcribed from the container label the name of the medication, providers’ information, expiration date, strength of the drug, instructions, special warnings, etc. The medication assessment of this study employed the methodology established by Sorensen and colleagues [39–41], and has been adopted by our research team previously [42–47].

#### Migraine Headache

Migraine was measured using self-reported data. They were asked, “have you ever been diagnosed with migraines by a health care provider?” Responses were yes and no.

### Data Analysis

Our analysis was divided into three parts. The first part was a descriptive analysis of all participants, reported as means and standard deviation for continuous measures and frequency and percentages for the categorical variables. Second, we conducted chi-square test, independent *t*-test, and ANOVA to examine the bivariate association between socio-demographic variables, and a few other relevant variables and migraine. Finally, we used multivariate generalized linear models (GLM) with Poisson distribution and log link; multiple linear regression; and multiple logistic regression to examine the independent association of migraine on various outcomes. Controlling for age, gender, education, marital status, type of insurance, satisfaction with and access to medical care, and the number of major chronic conditions, association between migraine, and several outcome variables were examined. For GLM and logistic regression, Odds Ratio (OR) and 95% confidence intervals are reported. For linear regression, standardized beta coefficients are reported. For multivariate analysis, *p*-values of less than 0.05 were considered significant. The Bonferroni correction methods were used to counteract the problem of multiple comparisons at the bivariate association.

## Results

### Univariate Analysis

Table 1 reports the characteristics of the study sample. This study included 740 African American individuals who were between the ages of 55 and 95 years (mean = 71.73 ± 8.37). Approximately 35% of participants were 75 years of age or older, with only 15% self-reported being married. One out of four participants never completed high school. Regarding health status, we noted the following health conditions/illnesses: diabetes mellitus (35%), hypertension (89%), heart-related conditions (30%), and COPD (26%).

**Table 1:** Characteristic of sample and bivariate association between migraine headache and other related outcomes (*n* = 740)

Sample characteristics	Total sample	Migraine headache		Sig
		No	Yes	
	*N* (%) Mean ± SD	*N* (%) Mean ± SD	*N* (%) Mean ± SD	
*Gender*				0.021
Male	266 (36)	226 (85)	40 (15)	
Female	472 (64)	371 (79)	101 (21)	
Age				0.002
55–64	120 (16)	80 (67)	40 (33)	
65–74	359 (49)	302 (84)	57 (16)	
75 and older	260 (35)	214 (83)	44 (17)	
*Education*				0.084
No high school diploma	183 (25)	143 (79)	39 (21)	
High school diploma	265 (36)	207 (78)	58 (22)	
Some college/graduate	292 (39)	247 (85)	44 (15)	
*Married/partner*				0.113
No	627 (85)	505 (81)	121 (19)	
Yes	113 (15)	92 (82)	20 (18)	
*Disability status*				0.087
No	473 (64)	406 (86)	65 (14)	
Yes	267 (36)	191 (71)	76 (29)	
Sleep disorder				0.000
No	541 (73)	462 (85)	79 (15)	
Yes	197 (27)	135 (69)	62 (31)	
	Mean ± SD	Mean ± SD	Mean ± SD	Sig.
Financial strains (rarely: 1 – always: 5)	1.83 ± 1.23	1.74 ± 1.07	2.24 ± 1.28	0.000
Physical health quality of life	40.26 ± 12.23	41.60 ± 12.06	34.51 ± 11.34	0.000
Mental health quality of life	52.28 ± 10.92	53.22 ± 10.13	48.37 ± 13.16	0.000
Self-rated health	3.13 ± 1.02	3.05 ± 1.00	3.48 ± 1.01	0.000
Severity of pain	2.05 ± 2.25	1.72 ± 2.02	3.47 ± 2.62	0.000
Major chronic conditions (0–6)	2.11 ± 1.10	2.07 ± 1.08	2.31 ± 1.15	0.019
Depressive symptoms	2.47 ± 2.77	2.10 ± 2.46	4.06 ± 3.44	0.000
Number of office-based provider visits	5.51 ± 3.27	5.48 ± 3.29	5.67 ± 3.20	0.543
Number of ED admissions	0.79 ± 1.58	0.69 ± 1.39	1.22 ± 2.18	0.000
Number of hospital admissions	0.54 ± 1.33	0.54 ± 1.40	0.54 ± 0.99	0.984
Number of Rx used	5.87 ± 3.24	5.68 ± 3.22	6.67 ± 3.23	0.002
Number of OTC used	1.18 ± 1.93	1.19 ± 1.86	1.14 ± 2.19	0.797

### Bivariate Analysis

Table 1 also shows bivariate correlations between migraine and other related variables based on the chi-square, *t*-test, and ANOVA F-test. Women, young-old, disabled participants, those with higher level of financial strain reported more migraine than their counterparts. In addition, association between reporting migraine and following variables were detected: lower level of HRQoL, higher number of depressive symptoms, sleep disorder, higher number of Rx medication use, higher number of ED admissions, and lower level of self-reported health status. No association between, migraine and office-based provider visits, hospital admissions, OTC use, marital status, or education was detected.

### Multivariate Analysis

Table 2 contains the condensed results of 12 independent multivariate analyses. Each analysis controlled for age, gender, education, marital status, financial strain, insurance type (Medicare and Medi-Cal), satisfaction with and access to medical care, and the number of major chronic conditions while examining the independent association between migraine and 12 outcome variables. Having migraine was associated with three categories of outcomes: (1) higher level of health care utilization measured by (i) ED admissions, and (ii) number of medication use; (2) lower level of HRQoL and health status measured by (i) lower self-rated health, (ii) physical QoL, and (iii) mental QoL; and (3) worse physical and mental health outcomes measured by (i) higher number of depressive symptoms, (ii) higher level of pain, (iii) sleep disorder, and (iv) being disabled.

**Table 2:** Multivariate analysis of association between migraine headache and outcome variables among underserved older African American adults. Adjusted for age, gender, education, financial strains, access and satisfaction with care, and number of chronic conditions (*N* = 740)

Dependent/outcome variable	Model	Independent variable: migraine headache (No vs Yes)					
		B	Beta	95% CI unstandardized coefficients	Exp. (B) OR	95% CI Exp. (B) OR	Sig.
Health care utilization							
Physician visits	MLR	−.113	−.014	−.718–.492	-	-	.714
ED admissions	GLM-P	.357	-	-	1.429	1.182–1.728	.000
Hospital admissions	GLM-P	−.176	-	-	.839	.647–1.088	.185
Number of Rx used	MLR	.687	.083	.127–1.247			.016
Number of OTC used	GLM-P	.060	-	-	1.062	.886–1.274	.516
Quality of life and perceived health							
Physical health quality of life	MLR	−4.225	−.135	−6.321–−2.128	-	-	.000
Mental health quality of life	MLR	−3.053	−.110	−4.979–−1.127	-	-	.002
Self-rated health	MLR	.249	.096	.068–.430	-	-	.007
Health outcomes							
Depressive Symptoms	MLR	1.297	.183	.842–1.751	-	-	.000
Severity of pain	LT-SLR	.377	.215	.262–.493	-	-	.000
Sleep disorder							.001
No	BLR	.704	-	-	1.00	Ref	
Yes					2.021	1.336–3.057	
Disability status							.003
No	BLR	.639	-	-	1.00	Ref	
Yes					1.895	1.241–2.893	

*MLR* multinomial logistic regression, *GLM-P* generalized linear regression with Poisson distribution,* LT-SLR* log transferred linear regression, *BLR* binary logistic regression

## Discussion

Of the older African American adults who participated in our study, more than 21.4% of women and 15% of men (55 years and older) reported migraine headaches. We searched NHANES, BRFSS, and CHIS datasets and found the prevalence of migraine headaches only in NHANES 1999–2004 for adults by race and age. The prevalence of migraines for White men and women were 8.0% and 14.3% and for Black men and women (for the same age group) were 11.7% and 16.4%, respectively. Our findings show a much higher prevalence of migraine in our sample of underserved African Americans aged 55 years and older compared with the NHANES data for the same age group of White and Black women and men. Our survey and the NHANES used a similar item to measure self-report of migraine. The NHANES survey asked “During the past 12 months, did you have severe headaches or migraines?” whereas, our survey asked “Have you ever been diagnosed with migraines by a health care provider?” [48].

### Migraine and Health Care Utilization

Our findings indicate that older African American adults who suffer from migraine headaches utilized health care services more often than non-migraineurs, reflecting the contribution of comorbidities and help-seeking behavior. Our study shows that 54% of African Americans 55 years and older who suffered from migraines used ED services at least once within the past 12 months prior to the interview. Indeed, those who reported being diagnosed with migraine had 100% increased odds of using ED services compared with their counterparts with no migraine. Examination of 11 years of data from the National Ambulatory Medical Care Survey showed that the uninsured, and those with Medicaid, receive substandard therapy for migraine, mostly because they received more care in EDs and less in physicians’ offices [49]. This is demonstrated in our results, as there was no increase in care received in physician offices among migraine patients compared to non-migraine patients. To review the scope of the challenges that vulnerable populations are facing, Charleston and colleagues in 2018 argued that underrepresented minority patients encounter numerous healthcare barriers, including lack of access, insurance coverage of treatment, transportation to and from appointments, and prohibitive costs [18]. A recent study conducted by Sheikh and colleagues in 2020 examining predictive factors for pain-related ED returns in middle-aged and older adults, revealed that African Americans who return to the ED for pain have low health literacy [50].

An extensive review of the literature shows that migraine patients do not get the appropriate recommended migraine-specific medications in the ED and instead receive opioids, which have greater risks of abuse and addiction [51]. This could be increasingly the case in older adults whose nephrogenic and/or cardiovascular comorbidities, such as chronic kidney disease and coronary artery disease, limit their ability to receive medications such as triptans, ergot derivatives, and/or ketorolac, which are commonly used as abortive therapeutics for migraine in the ED. Another recent scoping review of literature by Lim and colleagues (2021) reported almost 90% of studies commenting on adherence to hospital or evidence-based guidelines stated that treatment practices for migraine in the ED are varied and stray from established guidelines [52]. In addition, there is empirical data indicating that ED visits commonly result from an inability to access care elsewhere or missed opportunities for diagnosis and treatment [53]. Using the New York City Department of Health and Mental Hygiene Syndromic Surveillance database, Minen and colleagues(2018) conducted a retrospective nested cohort study from 18 NYC EDs and documented that more than 25% of individuals who make an initial ED visit for migraine pain will return to the ED within the next 6 months for another migraine-related visit [54]. Improving migraine-related care for underserved minority older adults in the ED in underserved communities requires coordinated efforts on the part of emergency medical providers, primary care providers, and public health practitioners to address factors related to the overuse of ED services by older minority adults. Challenges and barriers that affect underserved older African American adults with migraine cannot be fully addressed in an over-crowded ED.

Our data further showed that there was no significant increase in hospital admissions among older African American adults with migraines in the past 12 months that the interview occurred. This was an expected finding. While a debilitating and disabling condition, migraine pain rarely requires hospital admission and inpatient care except in the case of intractable vomiting and medication over-use due to treatment with opioids and/or barbiturates [55, 56].

There was no significant difference in the number of office-based provider visits among study participants with migraine compared to non-migraineurs. This finding may coincide with the similar number of major chronic health conditions among non-migraineurs and migraineurs that participated (2.01 and 2.37 respectively). Approximately 70% of patients with migraine that are followed by a clinician for their headache disorder are treated by primary care providers, with the remainder of patients seen by a neurologist or pain specialist [57]. Given that the vast majority of migraine patients see their primary care provider, the patient may elect to discuss migraine-related concerns at annual visits or other pre-planned follow-up visits rather than migraine-specific appointments.

### Migraine and Medication Use

Our results demonstrate a strong association between migraine and the use of prescription medications. The number of over-the-counter medications was similar between those with migraine and non-migraineurs (1.14 and 1.19, respectively). However, migraine sufferers used six prescription medications compared to five prescriptions among non-migraineurs.

The options for abortive and preventive migraine therapeutic options are expansive and drug choice is often dependent on the comorbidities of the patient and concurrent pharmacotherapy use. Available medication options become further constricted in older adults as the incidence of comorbidities and potential for drug interactions rise, particularly for abortive medications where the risk of gastrointestinal, cardiovascular, and renal side effects is more prevalent [58]. The American Headache Society recommends optimizing migraine drug selection with consideration to comorbidities and concurrent medication usage [58]. Unfortunately, older adults are frequently excluded from new drug trials for novel migraine preventive medications, such as the calcitonin gene-related peptide monoclonal antibodies, which means safety data is often limited.

Over-the-counter migraine medications are primarily limited to abortive treatment for mild-to-moderate headache pain, such as acetaminophen-aspirin-caffeine combinations and non-steroidal anti-inflammatory drugs. Older patients with coexisting chronic kidney disease, gastrointestinal bleeding, and/or liver disease may not be the best candidates for frequent or repetitive use of these medications and may instead seek out their primary care provider or headache specialist for a prescription medication for preventive headache control or abortive relief [59, 60]. Alternatively, a migraineur may elect to utilize ED services in the event of a moderate-to-severe migraine, especially one who may not have a consistent patient-provider relationship.

### Migraine and Health-related Quality of Life

Our data revealed a strong association between migraine and low scores on the SF-12 Health Survey measuring mental health and physical health quality of life compared to other persons of the same age, with an average score of 35.51 and 48.37 out of 100 for physical and mental quality of life, respectively. Older African American adults with migraine who participated in our study also demonstrated poorer self-rated health status responses compared to participants who did not have migraine. A review of the literature revealed scarce studies on the effect of migraine on quality of life among minority older adults, yet numerous reporting the effect of various therapeutic interventions on quality of life in adults with migraines, including those investigating novel treatments and treatments considered alternative.

It is well known that migraine causes significant disability. The impact is not isolated to the pain during the headache attack but includes the associated disablement due to loss of function, failed treatment attempts, and reduced productivity. Patients with migraine may experience increased stress, absenteeism, reduced ability to participate in social activities, and increased costs accrued from health care visits and prescriptions compared to those who do not have migraine [61]. The negative impacts of migraine should be considered given its pathophysiological contribution to the development of comorbid conditions associated with migraine and the psychosocial influence on the patient [61]^.^

### Migraine and Depression

After adjusting for other relevant variables, our study showed an association between migraine and depression. Older African American adults with migraines in this study had higher scores on the geriatric depression scale, providing affirmative responses to an average of four GDS items. The association between migraine and the development of depression is well known [62]. This connection may be related to hypothalamic-pituitary-adrenal axis activation leading to a decline in serotonin during migraine headache. Specifically, in older adults, pain is a significant risk factor for the development of depressive mood [63]. The national Health and Retirement Study demonstrated two key findings in congruence with our study: (1) chronic pain is closely associated with the development of depressive symptoms, and (2) African Americans adults have greater pain-related disability than other adults with chronic pain [64]. This is particularly relevant given the high SF-MPQ-2 and GDS scores among our patients with migraines.

### Migraine and Sleep Disorder

Our data revealed a strong association between migraine and sleep disorders. There are few studies examining the presence of sleep disorders in older adult patients with migraine headaches, with no studies specifically looking at minority populations with migraine. These studies in older populations have noted the positive association between migraine and poor sleep [65]. Prior cohort studies have demonstrated an increased prevalence of restless leg syndrome (RLS) and poor sleep quality among migraine patients [62]. The sleep association does not end at RLS, however, as numerous other sleep disorders and parasomnias have been implicated, including insomnia [66]. Additionally, obstructive sleep apnea (OSA) and other sleep-disordered breathing have been found to be risk factors for the development of chronic daily headache [67]. In addition to comorbid disorders, sleep is frequently compromised and implicated with the migraine itself; with sleep deprivation often listed as a trigger for a migraine attack and sleep impairment often listed as a consequence of ongoing migraine pain [66].

### Migraine and Disability

When adjusting for age, gender, education, financial strain, healthcare access and satisfaction, and number of chronic health conditions, there was a significant association between disability status and migraine headache in our participants. Migraine is one of the leading causes of disability in the USA and the world [68]. Headache disorders, including migraine, have been ranked 14th worldwide among causes of disability-adjusted life years and 2nd worldwide for all ages and sex among causes of years lived with disability. While the worldwide analysis of disability-adjusted life years and years lived with disability were attributed to general headache disorders, 88.2% were attributed to migraine [69].

### Strengths and Limitations

The limitations of this study include the non-random convenient sample and cross-sectional design of the study, which limits its generalizability. Because of the cross-sectional design of our study, we cannot make any causal inferences. Second, we did not have access to objective medical histories and records for the participants; therefore, we relied on self-report of chronic conditions. For example, the assumption is made that participants brought in all of the medication vials that they used within 2 weeks prior to the interviews. Lack of access to participants’ medical or pharmacy records limits our ability to validate this assumption and isolate whether participants were prescribed any medication for migraine or whether emergency department visits were for treatment of migraine headache. Despite these limitations, this study and its findings provide valuable insight into the prevalence of migraine headache in older African American adults, associated health care utilization, and health–related outcomes. These results can represent an inflection point for future research addressing health care disparities related to migraine.

## Implications

This study documented a much higher prevalence of migraine headache in older African American adults than other national data of the same age group, which speaks to a greater health disparity among migraine than previously thought. Migraine headache significantly correlates with the quality of life, healthcare utilization, and many health outcomes among underserved African American middle-aged and older adults. After adjusting for other relevant variables, our study showed that there was an association between migraine and depression as well as sleep disorders. Similarly, this study documented a significant association between disability status and migraine headache. Our results also demonstrated a strong association between migraine and the use of prescription medications.

Given our study results, the high prevalence of migraine in this population and the association of migraine with disability, healthcare utilization, and comorbidities, there is increased suffering related to migraine headache among underserved African American older adults that extends far beyond the headache pain itself, as evidenced by reduced quality of life, disability status, and depression. Migraine headache is often thought of as a condition of young adults; our study brings awareness to the diagnosis and burden of migraine specifically in underserved African American older adults.

Migraine is a chronic condition requiring solutions targeted at chronic disease management. Health care providers working in underserved areas need to be aware of the diagnosis within this population and be cognizant of appropriate treatment options to manage headache within this population based on comorbidities to reduce disability associated with the disease and utilization of emergency medicine services. Specialists are often not needed to make the diagnosis of migraine and in under–resourced communities, waiting to get in with a specialist can significantly delay the diagnosis and initiation of treatment.

Diagnoses and treatments of migraine among underserved older African American adults require multi-faceted and culturally sensitive interventional studies. Further studies assessing the prevalence of migraine in older African American adults would be beneficial in addressing the true impact of migraine in this population.

## Data Availability

N/A.
